# A Rare Case of Müllerian Agenesis With a Giant Tumor Arising From Uterine Remnants

**DOI:** 10.1155/crog/4292888

**Published:** 2025-12-15

**Authors:** Natalia Milczarska, Ján Varga, Karolina Just, Alexander Ostró

**Affiliations:** ^1^ Department of Gynaecology and Obstetrics, Univerzita Pavla Jozefa Safarika v Kosiciach Lekarska fakulta, Košice, Slovakia

**Keywords:** case report, leiomyoma, MRKH syndrome, Müllerian agenesis, surgery

## Abstract

**Introduction:**

Müllerian agenesis, also known as vaginal agenesis, Müllerian aplasia, or Mayer–Rokitansky–Küster–Hauser (MRKH) syndrome, is a rare developmental disorder affecting one in 5000 female births, resulting from an error in Müllerian duct fusion during fetal development.

**Case Report:**

We present a clinical case of a 46‐year‐old female patient, virgo intacta, with Müllerian agenesis and a leiomyoma arising from remnant uterine buds. The patient presented with primary amenorrhoea, continuous lower abdominal pain, and abdominal distension. We describe the diagnostic process and treatment strategy.

**Discussion:**

MRKH syndrome is the leading cause of vaginal agenesis, often accompanied by uterine and cervical aplasia. It is categorized into Type 1, which involves symmetric underdevelopment of the Müllerian ducts, and Type 2, characterized by asymmetric malformations of the genital tract and other congenital anomalies. Leiomyomas arising from remnant uterine tissue in MRKH patients are extremely rare. Accurate diagnosis and a carefully planned treatment strategy are essential for managing patients with MRKH syndrome complicated by rare tumors.

**Conclusion:**

In this case, total tumor extirpation with bilateral adnexectomy was the preferred treatment, based on the patient′s clinical presentation and diagnosis.

## 1. Introduction

Müllerian agenesis, also referred to as vaginal agenesis, Müllerian aplasia, or Mayer–Rokitansky–Küster–Hauser (MRKH) syndrome, is a rare congenital malformation occurring in approximately one in 5000 female births [1, 2]. It is caused by the failure or incomplete fusion of the Müllerian ducts during embryonic development, which impairs the normal formation of the upper two‐thirds of the vagina, uterine cervix, and uterus [2, 3]. In contrast, the lower one‐third of the vagina arises from the urogenital sinus [4]. In patients with Müllerian agenesis, there is a characteristic formation of a fibromuscular cord resembling a hypoplastic bicornuate uterus, arising from the proximal ends of the thickened uterine tubes [5]. Despite significant research, the precise etiology of the disease remains unknown, although environmental and genetic factors are thought to contribute [5, 6]. MRKH syndrome is the second most common cause of primary amenorrhoea in phenotypically female individuals, following gonadal dysgenesis [2, 5, 6].

MRKH syndrome is characterized by a 46XX karyotype and normally functioning ovaries, leading to typical female secondary sexual characteristics, including normal breast development and external genitalia, along with normal female patterns of axillary and pubic hair [1, 7].

MRKH syndrome is classified into two subtypes based on anatomical differences and associated anomalies [2, 5].

1. Type 1 (typical MRKH): This variant, which comprises approximately 44% of all MRKH cases, is marked by symmetric underdevelopment of the caudal portions of the Müllerian ducts. It manifests as the complete aplasia of the upper two‐thirds of the vagina and cervix, as well as the presence of symmetric muscular uterine buds with structurally normal fallopian tubes [2, 3, 7].
2. Type 2 (atypical MRKH): The atypical form, accounting for about 56% of MRKH cases, is characterized by asymmetric hypoplasia of one or both Müllerian ducts, leading to formation of asymmetric muscular uterine buds and abnormal fallopian tubes. Along with vaginal, cervical, and uterine aplasia, individuals with Type 2 MRKH syndrome frequently present with additional anomalies affecting the urogenital tract, skeletal system, or auditory apparatus. Some cases present with agenesis of the entire vagina and cervix, along with rudimentary uterine structures and dysmorphic fallopian tubes [2, 3, 7].

Moreover, in some sources, there is also described the third type of MRKH syndrome, called MURCS type–Müllerian aplasia, renal aplasia, and cervicothoracic somite dysplasia [5, 7].

The uterine remnants in MRKH syndrome may contain variable amounts of myometrial tissue, which may or may not include an endometrial lining or uterine cavity [5]. When endometrial tissue is present, it can lead to conditions such as hematometra, endometriosis, or adenomyosis, further complicating the clinical picture with cyclic abdominal pain and abdominal distention [1, 4, 6]. Very rarely, residual myometrial tissue within a rudimentary uterus can give rise to leiomyomas or fibroids. These leiomyomas, when present, may form significant abdominal masses, leading to symptoms such as pain, abdominal distention, and discomfort [2, 3].

Here, we present a case of a giant leiomyoma arising from uterine remnants in a patient with Type 1 MRKH syndrome.

## 2. Case Report

**Figure 1:**
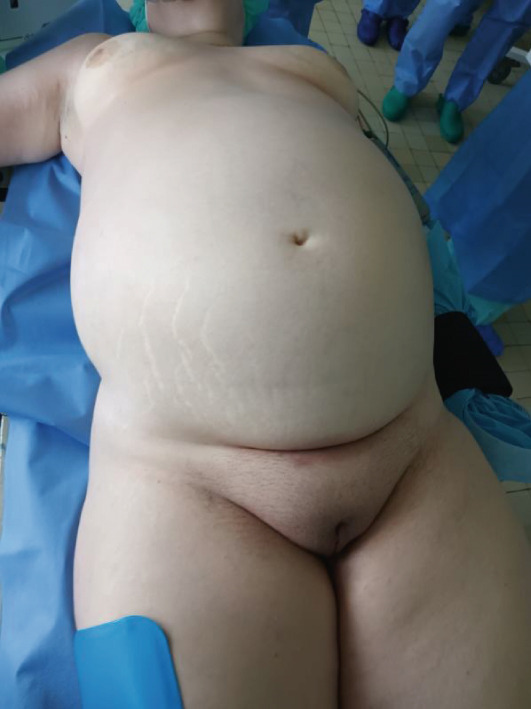
Preoperative view of the patient′s distended abdomen with visible striae. Normal secondary sexual characteristics are also visible.

A 46‐year‐old female with a 46XX karyotype presented with a 2‐year history of progressive abdominal distention (Figure 1). Her medical history included primary amenorrhea, infertility, and diagnosed vaginal aplasia. The patient showed normal secondary sexual characteristics, with typical external genitalia, breast development, and hair pattern. The patient′s family history was unremarkable. She denied any medical history of cancer, cardiovascular, endocrine, genitourinary, gastrointestinal, psychiatric, or blood disorders.

Upon admission, she was consulted by a gynaecologist. Physical examination revealed no abnormalities at the external urethral orifice or external genitalia; however, the vaginal canal was obliterated, preventing uterine sound insertion. Additionally, a smooth, mobile mass was palpable upon rectal examination.

The patient underwent complete preoperative evaluation, including abdominal ultrasound; CT of the chest, abdomen, and pelvis; complete blood count (CBC); comprehensive metabolic panel (CMP); genetic examination; tumor markers (CA 125, HE4, ROMA index, CA 72‐4, CA 19‐9, CEA, AFP, and HCG); CRP; and coagulation studies. Results were largely unremarkable, though uric acid was elevated at 476 *μ*mol/L (normal: 142–339 *μ*mol/L).

**Figure 2:**
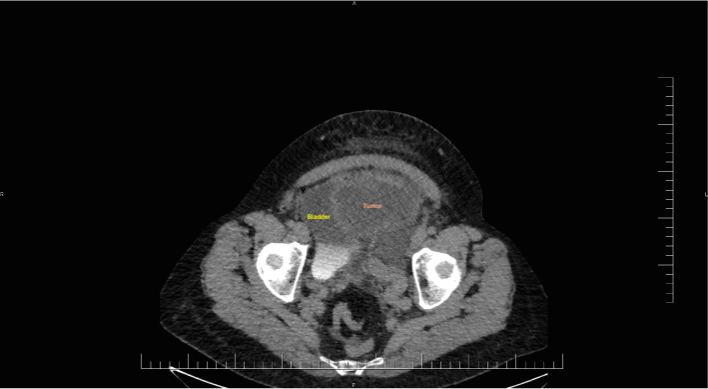
Axial CT image showing the close anatomical relationship between the urinary bladder and the tumor.

Abdominal ultrasound revealed a heterogeneous mass in the mesogastrium and hypogastrium, of unclear origin, with suspected uterine myoma and free abdominal fluid. CT imaging identified a 30 × 20 × 32 cm nonhomogenous mass in the pelvic and abdominal cavities, sharply demarcated, likely of uterine origin. Paracolic structures consistent with displaced ovaries were visible (right: 36 mm and left: 25 mm), along with elongated ovarian vessels; in addition, lower pelvis varices were detected. CT imaging also demonstrated the close anatomical relationship between the urinary bladder and the tumor (Figure 2). Free abdominal and pelvic fluid was also noted. No other organ abnormalities were identified.

Preoperative urological and surgical assessments, including colonoscopy (which showed Grade II internal haemorrhoids) and urological ultrasound (with no urological anomalies identified), were completed with findings confirming suitability for surgery.

Moreover, the patient reported no prior attempts at nonsurgical vaginal elongation or vaginoplasty. The patient′s psychological evaluation was unremarkable.

**Figure 3:**
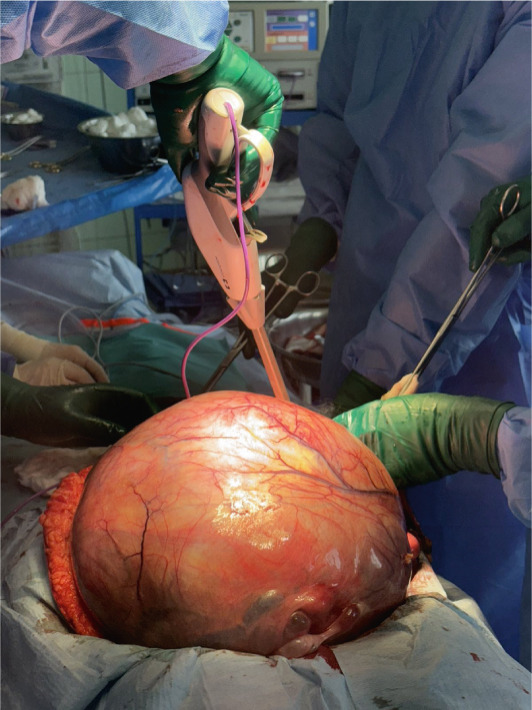
Operative exploration shows a large pelvic mass with a smooth capsule, closely adhering to the urinary bladder.

**Figure 4:**
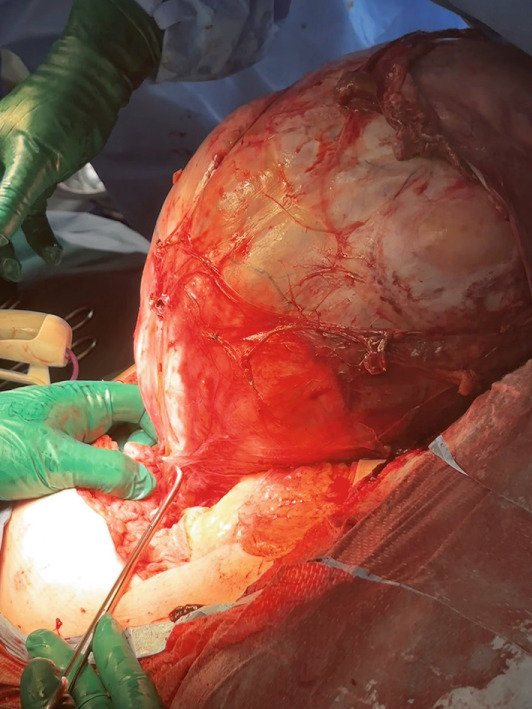
Operative exploration shows careful dissection of dense adhesions between the tumor and urinary bladder.

**Figure 5:**
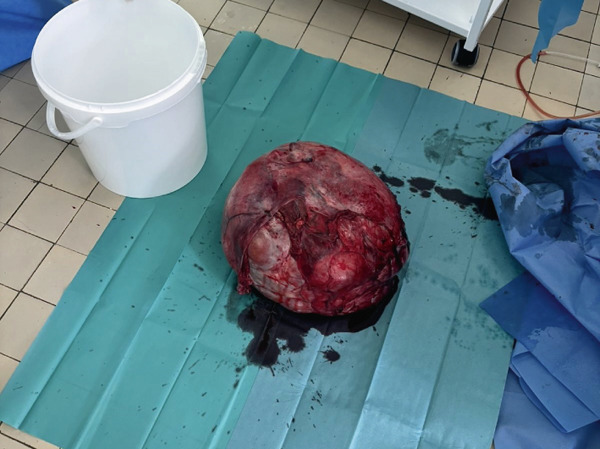
Image of the specimen after performing total tumor extirpation with bilateral adnexectomy.

Under general anesthesia, a midline laparotomy was performed, revealing a large, smooth‐capsule tumor (Figure 3) (32 × 28 × 27 cm) closely adherent to the urinary bladder (Figure 4), resulting in an iatrogenic bladder perforation during dissection. A total tumor extirpation with bilateral adnexectomy was performed (Figure 5). The intraoperative frozen section identified the tumor as a leiomyoma without atypia. The bladder perforation was repaired in two layers. Final histology revealed no uterine cavity or endometrial tissue in the specimen. Both ovaries were atrophic with epithelial cystic inclusions, while the fallopian tubes showed no pathology. The total operative time was 180 min, and the estimated blood loss was 650 mL. Postoperative management included two transfusions of packed red blood cells and one unit of fresh frozen plasma administered on the day of surgery, urinary catheterization for 10 days, and antibiotic therapy. The patient was discharged in stable condition on postoperative Day 10 for outpatient follow‐up. The postoperative course was uneventful, and convalescence proceeded without complications. At the follow‐up urological examination after discharge, findings were normal, and no further urological follow‐up was required.

## 3. Discussion

This case illustrates the unique presentation and challenges of a 46‐year‐old patient with a giant leiomyoma originating from uterine remnants in already diagnosed vaginal agenesis. While MRKH is most commonly associated with primary amenorrhea and the absence of a uterus and vagina, leiomyomas are extremely rare, making this case particularly significant.

MRKH syndrome is a congenital anomaly marked by the failure of Müllerian duct development [6]. It results in variable degrees of vaginal, cervical, and uterine agenesis, often classified into two subtypes [2, 3, 7]. This patient was diagnosed with typical MRKH syndrome, Type 1, uniquely complicated by a giant leiomyoma and total vaginal agenesis.

Although the incidence of Müllerian agenesis is relatively low, MRKH syndrome should remain a key differential diagnosis for patients presenting with primary amenorrhea and abdominal pain. Only a few cases of Müllerian agenesis were reported in the literature; the first was documented by Beecham and Skiendzielewski in 1977 in Pennsylvania, United States [8].

While typical symptoms are narrowed to primary amenorrhoea and vaginal aplasia, cases of Müllerian agenesis with hormonally responsive uterine remnants can lead to complications such as endometriosis, adenomyosis, or, more rarely, leiomyomas characterized by abdominal pain, distention and discomfort [1, 5, 6]. Uterine remnants can vary in size; histologically, they are composed of fibromuscular tissue, and sometimes of typical uterine tissues, myometrium, and endometrium [5, 9]. The pathogenesis of leiomyoma development in MRKH is similar to that of myomas arising in a normal uterus; it involves residual myometrial cells within Müllerian remnants that retain the potential for neoplastic growth, under the influence of ovarian estrogen [5]. Estrogen‐driven proliferation of myocytes and fibroblasts within the remnant uterine buds advises complete excision of both, leiomyomas, and adjacent uterine remnants, during surgery [3, 6]. Genetic predisposition may also play a role, alongside environmental factors, as well as chromosomal rearrangements in myocytes of remnant uterine tissue [5, 6].

In this case, the leiomyoma presented as a palpable mass with progressive abdominal distention, emphasizing the diagnostic challenge in MRKH patients who lack typical uterine structures. The diagnostic approach involved multimodal imaging, with ultrasound and CT revealing a heterogeneous mass likely of uterine origin. Although MRI (magnetic resonance imaging) is considered a gold standard modality for optimal visualization of genital and urinary tract malformations, which can also help to distinguish the tumor′s origin, logistical limitations prevented MRI in this case [1, 4, 10, 11]. Nonetheless, a comprehensive assessment was achieved with the available imaging modalities. The results of CT and ultrasound imaging sufficiently guided diagnosis and treatment planning, enabling surgical intervention without further delay. The identification of free abdominal fluid and displaced ovaries further underscored complex pelvic pathology, highlighting the importance of comprehensive imaging for MRKH patients, in whom masses may arise from atypical pelvic or abdominal sites.

Due to the anatomical abnormalities and adhesions often present in such cases, surgical management is challenging. In this patient, a laparotomic total tumor extirpation with bilateral adnexectomy was recommended due to the tumor′s size and the potential for symptomatic relief. The decision to extend the surgery to include bilateral oophorectomy was made due to technical difficulties in dissecting the ovarian tissue. Additionally, the macroscopic appearance of the ovaries was suspicious, showing multiple cystic lesions, further described in pathological findings. Complications, such as bladder injury, are not uncommon given the distorted anatomy and adhesions seen in MRKH and require careful intraoperative management by a skilled surgeon and thorough postoperative care.

In managing this case surgically, our primary objective was the excision of the large abdominal mass, which significantly impacted the patient′s quality of life. This involved the excision of all uterine remnants, fallopian tubes, and macroscopically changed ovaries to reduce the likelihood of recurrence.

Even though the patient did not express interest in pursuing sexual health interventions, it remains essential to provide sexual education for patients with MRKH syndrome [1]. Presenting available options, including vaginal dilation methods as the first‐line treatment, or surgical methods including the creation of a neovagina, is a crucial component of holistic care and should not be overlooked in the management plan [2, 4].

This case presents the clinical variability and diagnostic complexity of MRKH syndrome, especially when complicated by large tumors. It emphasizes the need for increased awareness and multidisciplinary management in patients with Müllerian agenesis who present with abdominal masses. Further research is needed to better understand the mechanisms behind leiomyoma development in MRKH and to optimize the diagnostic and therapeutic approaches for these patients.

## 4. Conclusion

In conclusion, this case report highlights an uncommon manifestation of Müllerian agenesis with a large leiomyoma originating from rudimentary uterine tissue. While MRKH syndrome typically presents with primary amenorrhea, along with agenesis of the vagina, cervix, and uterus, and normal secondary sexual characteristics, the development of a large pelvic mass underscores the necessity for comprehensive diagnostic evaluation in patients with Müllerian agenesis. Multimodal imaging with ultrasound and CT was essential in the diagnostic process, allowing timely surgical intervention despite the absence of MRI. This case underscores the importance of individualized diagnostic approaches and collaborative management in atypical presentations of MRKH syndrome.

## Ethics Statement

This study was conducted following the Ethical Principles of the Helsinki Declaration and national laws.

## Consent

Informed written consent was achieved from the patient.

## Conflicts of Interest

The authors declare no conflicts of interest.

## Author Contributions

N.M. and K.J. drafted and revised the manuscript. J.V. conducted the diagnostic process and performed the surgery. A.O. supervised the case management and approved the manuscript for submission.

## Funding

This work was supported by the Kultúrna a Edukacná Grantová Agentúra MŠVVaŠ SR, 10.13039/501100006108, 024SPU‐4/2023 012SPU‐4/2023.

## Data Availability

The data that support the findings of this study are available from the corresponding author upon reasonable request.
